# A Novel Restraint Device for Injection of *Galleria mellonella* Larvae that Minimizes the Risk of Accidental Operator Needle Stick Injury

**DOI:** 10.3389/fcimb.2017.00099

**Published:** 2017-03-28

**Authors:** James P. Dalton, Benedict Uy, Simon Swift, Siouxsie Wiles

**Affiliations:** ^1^Bioluminescent Superbugs Lab, University of AucklandAuckland, New Zealand; ^2^Department of Molecular Medicine and Pathology, University of AucklandAuckland, New Zealand; ^3^Maurice Wilkins Centre for Molecular BiodiscoveryAuckland, New Zealand

**Keywords:** *Galleria* grabber, infectious diseases, *Staphylococcus aureus*, caterpillar, bacteria

## Abstract

Larvae of the insect *Galleria mellonella* are increasingly being used for studying pathogenic microbes and their virulence mechanisms, and as a rapid model for screening novel antimicrobial agents. The larvae (waxworms) are most frequently infected by injection of pathogenic organisms into the haemocoel through the insect's prolegs. The mostly widely used method for restraining the waxworms for injection is by grasping them between the operator's fingers, which puts the operator at risk of needle stick injury, an important consideration when working with highly pathogenic and/or drug-resistant microorganisms. While use of a stab proof glove can reduce this risk of injury, it does so at the loss of manual dexterity and speed, resulting in a more labor-intensive, and cumbersome assay. We describe a simple cost effective device (the so-called “*Galleria* Grabber”) for restraining waxworms for injection that keeps the operator's fingers clear of the needle thus reducing the risk of injury.

## Introduction

Larvae (waxworms) of the Greater wax moth *Galleria melonella* have become a widely used surrogate host for studying pathogenic microbes. In recent years, they have been used for studying virulence mechanisms, investigating differences between clinical isolates as well as for preliminary investigation of the efficacy of antimicrobial compounds, for a wide range of both Gram-positive and Gram-negative bacteria (Joyce and Gahan, 2010; McLaughlin et al., 2012; Ramarao et al., 2012; Loh et al., 2013; Thomas et al., 2013; Williamson et al., 2014; Adamson et al., 2015; Champion et al., 2016; Johnston et al., 2016; Moreira et al., 2016; Nale et al., 2016; Yang et al., 2016), fungi (Alcazar-Fuoli et al., 2015; Forastiero et al., 2015; Borman et al., 2016; de Lacorte Singulani et al., 2016; Frenkel et al., 2016; Gago et al., 2016; Santos et al., 2016), and viruses (Garzon et al., 1978; Buyukguzel et al., 2007; Özkan and Coutts, 2015). The use of waxworms as a model host has many advantages. The waxworms themselves are cheap and easy to obtain from commercial insect suppliers, and can be housed in large numbers to allow for greater study sizes at low cost. Waxworms possess an innate immune system that contains many analogous functions to that seen in humans, including phagocytosis and the production of antimicrobial peptides and reactive oxygen and nitrogen species (Wojda, 2016). Unlike other non-mammalian model organisms, such as *Caenorhabditis elegans, Danio rerio*, and *Drosophila melanogaster* (Glavis-Bloom et al., 2012; Arvanitis et al., 2013; Panayidou et al., 2014; Lopez Hernandez et al., 2015), waxworms can be incubated at 37°C which allows for the study of clinically relevant human pathogens at a temperature that mimics the human host. Finally, as insects, *G. mellonella* are not currently subject to the same ethical restrictions that small mammalian models are, meaning there is a low barrier to entry for researchers wishing to move their studies into a model host.

Infection of waxworms is typically carried out on 5th instar insects, when the waxworms are at their largest, typically around 2 cm in length and 100 mg in weight. The most common method of infection is by injection into the haemocoel through the last proleg of the insect; methods for injection vary between laboratories. One method is to immobilize the needle itself and then place the waxworm onto the needle for injection. Another more favored method is to immobilize the waxworms between the operator's fingers (Fuchs et al., 2010) and place the needle into the insect's proleg, lifting the needle away from the operator with the insect attached before pushing the plunger on the syringe. Both of these injection techniques present a hazard to the researcher and can result in needle stick injury and possible infection.

A recent article highlighted the use of a stab-proof glove to reduce the chance of this type of injury while immobilizing the waxworms over a pipette tip fixed to some paper (Harding et al., 2013). We have tried this technique and found that, while safer for the operator, using a stab-proof glove reduces the efficiency of injection, from 3–4 to 1 infection per minute, resulting in a lower injection rate and a more labor-intensive assay. Because of this, we investigated the possibility of using a simple restraining device to hold waxworms in place for injection, in a way that removes the operator's hand from the vicinity of the needle, allowing for maximum mobility, and safety of the operator.

## Materials and methods

### Preparation of bacteria

The *Staphylococcus aureus* isolate XEN36 (Francis et al., 2000) (Perkin Elmer) was grown overnight with shaking at 200 rpm in Tryptic Soy broth (Oxoid) at 37°C. Cells were washed twice in phosphate buffered saline (PBS) (Sigma-Aldrich) and then resuspended in PBS to an optical density at 600 nm (OD_600_) of 1, equivalent to ~5 × 10^9^ CFU ml^−1^. Resuspended cultures were serially diluted and plated onto Tryptic Soy agar (Oxoid) to retrospectively determine the bacterial counts used for injection. Inoculation doses were drawn into 1 ml ultra-fine (29 gauge) needle insulin syringes (BD, Wellington) for injection into the waxworms. Groups of waxworms were injected with 20 μl of either phosphate-buffered saline (PBS) or ~5 × 10^7^, 5 × 10^8^, or 5 × 10^9^ CFU ml^−1^ *S. aureus* XEN36.

### Selection, infection, and monitoring of *G. mellonella* waxworms

**Figure 1:**
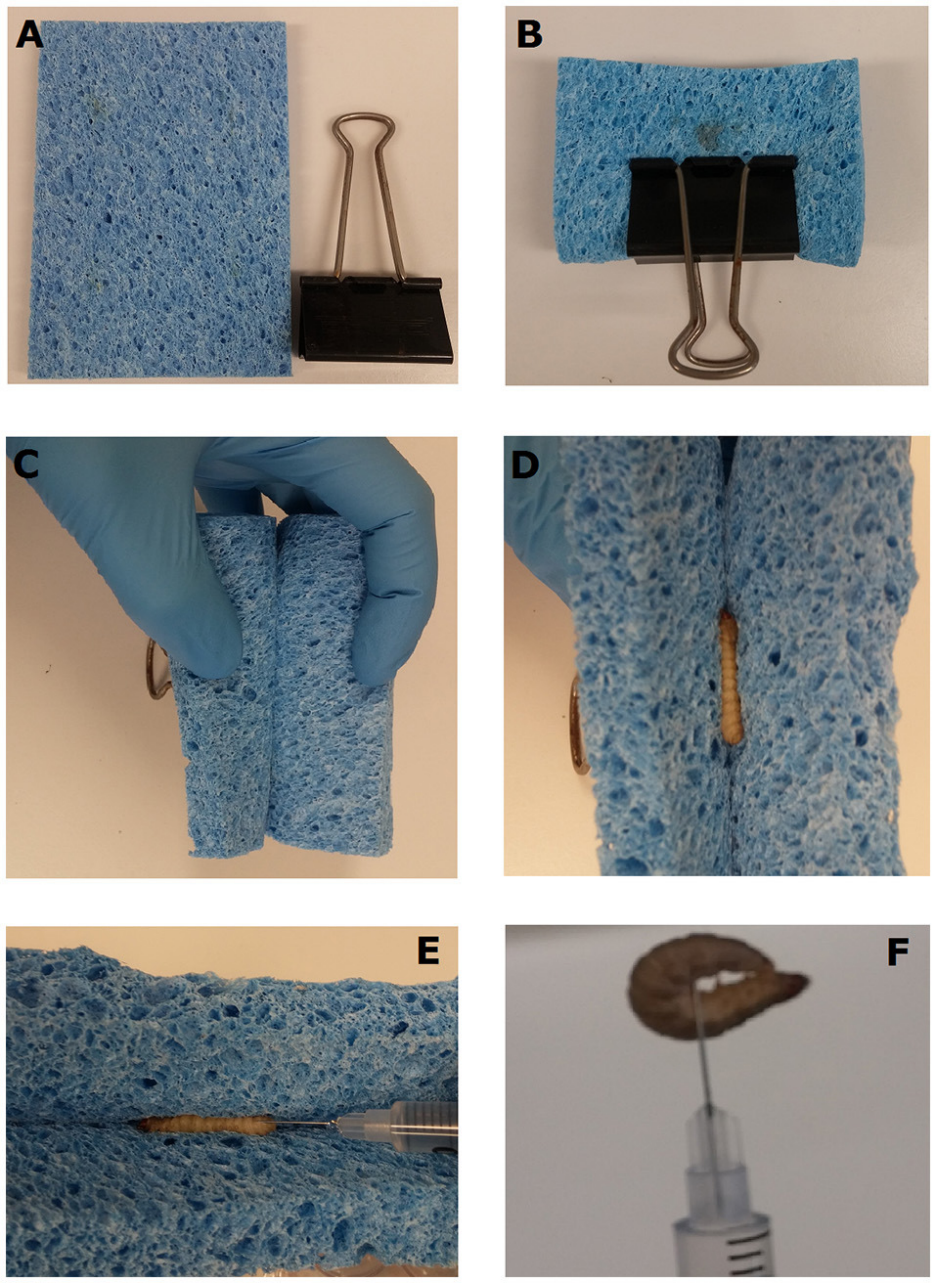
**Injection of waxworms using a novel restraint device**. The “*Galleria* Grabber” restraint device is comprised of a 15 mm thick sponge and bulldog clip (A). The sponge is folded in half lengthways and secured within a bull dog clip with the open end facing outwards **(B)**. The open ends of the folded sponge are peeled back and held in place **(C)**. The waxworm to be injected is placed within the sponge and held in place while the open end of the sponge is released. The closing of the sponge secures the waxworm in place for injection **(E)**. Once the needle is placed, the syringe is lifted with the waxworm in place and the plunger is pushed to inject the desired inoculum **(F)**.

Fifth instar waxworms were selected based on consistency in size and split into eight groups of 12. Four groups were injected with either PBS or doses of 10^6^–10^8^ CFU *S. aureus* XEN36 using the most common technique of grasping the waxworms between the operator's thumb and index finger and injecting into the waxworm's last proleg. The remaining four groups were injected with either PBS or doses of 10^6^–10^8^ CFU *S. aureus* XEN36 using the newly described restraining device (which we have dubbed the “*Galleria* Grabber”), which comprises a 12 × 9 cm kitchen sponge and a large bulldog clip (~50 cm) (Figure 1A). To comfortably restrain the waxworms, the sponge was folded in half and secured using the bulldog clip (Figure 1B). The open ends of the folded sponge were peeled back and held in place (Figure 1C). Next, a waxworm was placed within the sponge and held in place while the open end of the sponge was released (Figure 1D). Once the waxworm was securely held in place, the insulin syringe was inserted into the haemocoel via the insect's last proleg (Figure 1E). Once the needle was in place the waxworm was released from the restraining device (Figure 1F). If the needle is correctly placed, the waxworm remains attached to the needle of the syringe. Once the needle had been securely inserted into the waxworm, the insect was removed from the restraining device and the plunger of the syringe pushed down to inject the desired inoculum.

Once injected, waxworms were housed in individual wells of 24 well-tissue culture dishes (Nunc) with the lids taped down to ensure against escape. These dishes were placed inside a secondary container to ensure containment. Waxworm mortality was monitored over 5 days.

## Results and discussion

**Figure 2:**
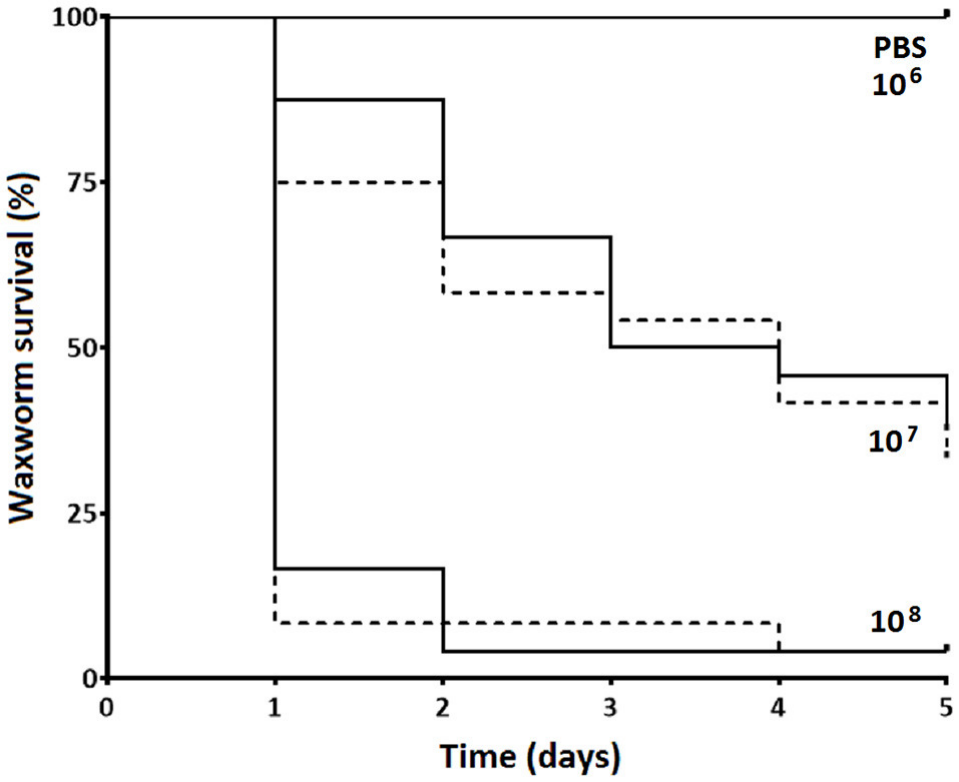
**Waxworms (*n* = 12 per group) were infected with varying concentrations of *S. aureus* XEN36 or phosphate-buffered saline (PBS) by injection into the haemocoel via the last proleg while restrained either between the thumb and index finger of the operator (solid lines), or using the “*Galleria* Grabber” restraint device (dashed lines), and survival measured over 5 days**.

We observed no differences in the infection dynamics between the groups of waxworms injected with *S. aureus* XEN36 after restraint using the novel “*Galleria* Grabber” device described compared to restraint by holding the waxworms between the operator's thumb and index finger. For both restraint techniques, we observed no mortality from the waxworms injected with PBS (Figure 2). In contrast, the majority of waxworms injected with ~10^8^ CFU *S. aureus* XEN36 died within 24 h (Figure 2). We observed a dose dependent mortality for waxworms injected with *S. aureus* XEN36, with 66% of waxworms injected with ~10^5^ CFU succumbing to infection (Figure 2). No mortality was seen after injection with 10^6^ CFU *S. aureus* XEN36 (Figure 2).

The “*Galleria* Grabber” allows for easy injection of a large number of waxworms (~3 per minute), while greatly reducing the opportunity for the operator to suffer a needle stick injury. With the increasing popularity of waxworms as a model host for studies involving dangerous human pathogens (Champion et al., 2016), including clinical and/or drug-resistant isolates, protecting researchers from accidental laboratory infection is of great importance. While the use of a stab-resistant glove addresses this issue, it does compromise the speed at which waxworms can be injected. With this new restraint method, we were also able to inject smaller waxworms with ease. Most importantly, the new methodology described removes the operator's hand from the vicinity of needles loaded with pathogenic/drug-resistant microbes, allowing for maximum mobility and safety of the operator without compromising the speed of the assay.

## Author contributions

JD, Conceived and designed the experiments; JD, BU, Performed the experiments; JD, SW, Analyzed the data; SS, Contributed reagents; JD, SW, Wrote the manuscript; JD, SW, Prepared the figures; JD, BU, SS, SW, Reviewed drafts of the paper.

## Funding

This work was supported by a University of Auckland new staff grant to SW (9802 3707601).

### Conflict of interest statement

The authors declare that the research was conducted in the absence of any commercial or financial relationships that could be construed as a potential conflict of interest.
